# A survey of *FLS2* genes from multiple citrus species identifies candidates for enhancing disease resistance to *Xanthomonas citri* ssp. *citri*.

**DOI:** 10.1038/hortres.2016.22

**Published:** 2016-05-11

**Authors:** Qingchun Shi, Vicente J Febres, Jeffrey B Jones, Gloria A Moore

**Affiliations:** 1 Horticultural Sciences Department, University of Florida, Gainesville, FL 32611, USA; 2 Plant Molecular and Cellular Biology Program, University of Florida, Gainesville, FL 32611, USA; 3 Plant Pathology Department, University of Florida, Gainesville, FL 32611, USA

## Abstract

Pathogen-associated molecular patterns (PAMPs)-triggered immunity (PTI) is an important component of plant innate immunity. In a previous study, we showed that the PAMP flg22 from *Xanthomonas citri* ssp. *citri* (Xflg22), the causal agent of citrus canker, induced PTI in citrus, which correlated with the observed levels of canker resistance. Here, we identified and sequenced two bacterial flagellin/flg22 receptors *(FLS2-1* and *FLS2-2)* from ‘Duncan’ grapefruit (*Citrus paradisi, CpFLS2-1* and *CpFLS2-2*) and ‘Sun Chu Sha’ mandarin (*C. reticulata*, *CrFLS2-1* and *CrFLS2-2*). We were able to isolate only one *FLS2* from ‘Nagami’ kumquat (*Fortunella margarita, FmFLS2-1*) and gene flanking sequences suggest a rearrangement event that resulted in the deletion of *FLS2-2* from the genome. Phylogenetic analysis, gene structure and presence of critical amino acid domains all indicate we identified the true FLS2 genes in citrus. *FLS2-2* was more transcriptionally responsive to Xflg22 than *FLS2-1*, with induced expression levels higher in canker-resistant citrus than in susceptible ones. Interestingly, ‘Nagami’ kumquat showed the highest *FLS2-1* steady-state expression levels, although it was not induced by Xflg22. We selected FmFLS2-1, CrFLS2-2 and CpFLS2-2 to further evaluate their capacity to enhance bacterial resistance using *Agrobacterium*-mediated transient expression assays. Both FmFLS2-1 and CrFLS2-2, the two proteins from canker-resistant species, conferred stronger Xflg22 responses and reduced canker symptoms in leaves of the susceptible grapefruit genotype. These two citrus genes will be useful resources to enhance PTI and achieve resistance against canker and possibly other bacterial pathogens in susceptible citrus types.

## Introduction

Citrus is a worldwide fruit crop with high value for both fresh and processed fruit markets. Unfortunately, bacterial diseases, such as citrus canker (*Xanthomonas citri* ssp. *citri* (*Xcc*)) and Huanglongbing (*Candidatus* *Liberibacter asiaticus*) have been damaging or even destructive to citrus production, leading to significant reduction in fruit quality and quantity that corresponds to heavy economic losses. Genetic resistance to citrus canker has been identified from citrus types including kumquats (*Fortunella* spp.) and mandarins (*Citrus reticulata* Blanco), although major commercially grown citrus types such as sweet oranges (*C. sinenesis* Osback), grapefruits (*C. paradisi* Macf.) and lemons (*C. limon* (L.) Burm. f.) are susceptible.^1–4^ Conventional breeding using disease-resistant sources can be lengthy; however, identification of specific disease-resistant genes will accelerate this process through marker-assisted selection or direct introduction of genes by genetic transformation.

Plants have two types of innate immunity inducible by pathogens.^5^ One is through the perception of pathogen-associated molecular patterns (PAMPs) mediated by the host’s pattern recognition receptors (PRRs), which trigger a series of defense responses including an oxidative burst,^6^ callose deposition,^7^ cascade induction of mitogen-activated protein kinases (MAPK)^8^ and induction of defense-associated genes.^9^ The consequences of these responses are stalled pathogen multiplication and disease development (PAMP-triggered immunity or PTI).^10^ The other type of immunity is achieved by recognition of specific pathogen effectors by the plant’s resistance proteins, resulting in a hypersensitive response and disease resistance to the pathogen relying on the specific effector for virulence (Effector-triggered immunity).^11^

PTI, as the first layer of defense, is important for the protection of plants.^5^ It also has the advantage of being broad-spectrum because it is triggered by PAMPs that are conserved among pathogens.^12^ FLS2, the receptor of bacterial flagellin,^13,14^ is an extensively studied PRR. It has been shown that *Arabidopsis* FLS2 (AtFLS2) is involved in resistance to both non-host^15^ and pathogenic bacteria.^10^ Based on research with model plants, sensitivity of flg22 and of other PAMPs has been used to evaluate level of resistance to important pathogens in crop plants including tomato,^16^ soybean^17^ and oilseed rape.^18^ Direct transformation of an exotic PRR can also confer resistance in the recipient plant. For example, interfamily introduction of the *Arabidopsis* EFR, a PRR for the perception of bacterial elongation factor Tu (EF-Tu), into tomato establishes sensitivity to EF-Tu and induces higher disease resistance to a range of pathogens containing this PAMP.^19^ Transferring XA21 from wild rice into cultivated species confers resistance to multiple *X. oryzae* pv. *oryzae* isolates.^20,21^ Furthermore, transgenic citrus plants expressing an FLS2 from *Nicotiana benthamiana* showed elevated responses to flg22 and reduced susceptibility to citrus canker.^22^ These results demonstrate the possibility of engineering disease resistance using PRRs from resistant species.

In a previous study, we established that there was a correlation between robust Xflg22 responsiveness and citrus canker resistance, which was manifested as extensive induction by Xflg22 of defense-associated genes and high reactive oxygen species (ROS) production in resistant citrus types but not in susceptible ones.^23^ Here we propose that the observed phenotypic variation in Xflg22 responses among different citrus species is mediated by the receptor FLS2, where differences in its function at the protein and/or transcriptional level result in the observed variations in the PTI response and the final outcome of the disease. Facilitated by the available citrus genomic databases, we identified citrus *FLS2* orthologs (*FLS2-1* and *FLS2-2*) based on BLAST searches using AtFLS2 as the query. A recent study in citrus reported one *FLS2* ortholog, termed *CsFLS2,*^22^ which is the *FLS2-1* characterized here. We focused on comparisons between *FLS2-1* and *FLS2-2*, and between citrus species with different canker resistance levels. The predicted protein sequences of FLS2s from ‘Nagami’ kumquat, ‘Sun Chu Sha’ mandarin and ‘Duncan’ grapefruit were examined. Real time PCR analysis indicated *FLS2-2* was more transcriptionally responsive to Xflg22 than *FLS2-1*, and the expression level of *FLS2-2* correlated with canker resistance. ‘Nagami’ kumquat had the highest *FLS2-1* steady-state expression among the citrus species studied. In addition, we show that transient expression of the candidate genes from resistant ‘Nagami’ kumquat (FmFLS2-1) and ‘Sun Chu Sha’ mandarin (CrFLS2-2) were able to enhance the Xflg22 response and reduce citrus canker symptoms in the highly susceptible ‘Duncan’ grapefruit. The two identified PRR genes have the potential of being a valuable resource for the production of cisgenic citrus plants with improved canker resistance while at the same time having high public acceptance, since the gene sequences are citrus derived.

## Materials and methods

### Plant material

All citrus plants used in this study were grown in pots under greenhouse conditions. Fully expanded leaves were collected for the DNA extractions to amplify the *FLS2* candidate genes. For the RNA extractions to study gene expression, RACE PCR and for the *Agrobacterium*-mediated transient expression assays, the plants were pruned 4–6 weeks before the experiments and young fully expanded leaves were employed.

### PCR amplification, cloning and sequencing of citrus *FLS2* genes

Genomic DNA was isolated with the DNeasy Plant Mini kit (QIAGEN, Gaithersburg, MD, USA) following the manufacture’s protocol, and used as the template for the PCR. PCR reactions were performed with Advantage 2 Polymerase Mix kit (Clontech, Mountain view, CA, USA). The primer pair VF397-VF399 was used to amplify *FLS2-1*, and VF395-VF396 to amplify *FLS2-2* (Supplementary Table 2). The PCR products were purified using either a QIAquick PCR purification kit or QIAquick gen extraction kit (QIAGEN) and subsequently cloned into pGEM-T Easy vectors (Promega, Madison, WI, USA) and sequenced.

For the amplification of *FLS2* candidates using rapid amplification of complementary DNA (cDNA) ends (RACE), total RNA was extracted using TriZol reagent (Invitrogen, Carlsbad, CA, USA) according to the manufacturer’s instructions followed by DNase treatment and cleanup with the RNeasy Plant Mini Kit (QIAGEN). The cDNA was synthesized with the SuperScript III Reverse Transcriptase kit (Invitrogen) with Oligo dT primers.

### Gene expression analysis

The cDNA synthesis reactions were performed using 1 μg of total RNA and M-MLV reverse transcriptase (Invitrogen) with random decamers.

Gene expression was measured by quantitative reverse transcription–PCR (RT–qPCR) using a StepOnePlus instrument (Applied Biosystems, Foster City, CA, USA). The reactions were set to comparative C_T_ (ΔΔC_T_) with fast amplification (95 °C, 20 s and 40 cycles of 95 °C 1 s, 60 °C 20 s). TaqMan MGB probe, primers and fast universal PCR master mix were all from Applied Biosystems and used for target sequence amplifications from 5 ng of cDNA (Supplementary Table 3). Amplification of 5.8S RNA (150 nm of 4, 7, 2′-trichloro-7′-phenyl-6-carboxyfluorescein (VIC)-labeled probe and 250 nm of each primer) were used as endogenous controls. The data obtained (relative quantitation, RQ) was first subjected to a *Q*-test^24^ for evaluation of outliers among replications and subsequently analyzed with JMP Genomics 5.0 (SAS Institute, Cary, NC, USA) for model fitting of standard least square means and Student’s *t*-test statistical significance analysis (*P*<0.05). The gene expression measurements were based on leaf samples from three replicates and all the experiments were repeated at least twice.

### *Agrobacterium*-mediated transient expression of citrus FLS2 candidates

The full-length protein-coding citrus FLS2 candidate genes cloned into pGEM-T Easy vector were sub-cloned into a plant expression vector (pUC118/FMV) driven by a 34S Figwort mosaic virus (FMV) constitutive promoter.^25^ The *FMV::FLS2s* cassettes were further sub-cloned into pCAMBIA2201 vectors and transformed into *Agrobacterium* strain AGL1 for the transient expression experiments.

A loop of freshly grown *Agrobacterium* containing the FLS2 expression constructs were incubated with shake (28 °C, 220 r.p.m.) overnight in YEP broth medium (5 g L^−1^ bacto beef extract, 1 g L^−1^ bacto yeast extract, 5 g L^−1^ bacto peptone, 5 g L^−1^ sucrose and 0.5 g L^−1^ MgSO4, pH 7.2) that contained the proper antibiotics. The bacterial culture was centrifuged at 4000 r.p.m. for 15 min and the bacterial pellet resuspended in inoculation medium (1.98 g L^−1^ magnesium chloride, 0.98 g L^−1^ 2-(*N*-morpholino) ethanesulfonic acid (MES), and 0.029 g L^−1^ acetosyringone, pH 5.3) and adjusted to an OD600 of 0.3–0.8. *Agrobacterium* preparations were infiltrated into the abaxial surface of leaves using a 1 cc syringe with a needle until half of the leaf was saturated. Infiltration with *Agrobacteria* containing the empty pCAMBIA2201 plasmid was used as the negative control. Leaf tissue was collected 3 days after the infiltration for transient gene expression analysis (β-glucuronidase histochemical staining assay (GUS assay) and RT–qPCR).

### GUS assay

*Agrobacterium-*infiltrated leaf tissue was tested for the expression of the GUS reporter gene (present in the pCAMBIA2201 plasmid). Small leaf segments were cut and placed into 96-well plates with 30 μl of GUS staining solution (80 mm sodium phosphate (pH 7.0), 0.4 mm potassium ferricyanide, 0.4 mm potassium ferrocyanide, 0.8 mm EDTA, 0.8 mg mL^−1^ X-gluc, 0.05% Triton X-100 and 25% ethanol (volume/volume)). The plate was vacuum infiltrated (60 cm Hg) for 10 min and incubated at 37 °C overnight. Fifty microliters of 95% ethanol/glacial acetic acid (3:1 volume/volume) was added to each well and the plate was incubated for 30 min at room temperature. The histochemical staining (blue staining) was recorded under an optical microscope.

### Oxidative burst assay

Leaf discs of 3.8 mm in diameter were obtained and kept in 150 μl of sterile water overnight in a 96-well plate at room temperature. The next day the water was replaced with 100 μl of assay solution (100 μm of luminol, 10 μg mL^−1^ of horseradish peroxidase and 100 nm of Xflg22). Light emission (relative light unit) was measured in 5-min intervals for 60 min using a luminescence microplate reader (BioTek, Winooski, VT, USA). Means and s.e. were calculated based on two independent experiments (*n*=15).

## Results

### Homologous search of citrus *FLS2* identified two candidate genes in the genomes of *C. sinensis* and *C. clementina*

There are citrus genomic databases publicly available for *C. sinensis* and *C. clementina.*^26,27^ Using the FLS2 protein sequences from *Arabidopsis* (AtFLS2, AT5G46330 or GI: 42568348), tomato (LeFLS2, GI: 723675671) and grapevine (VvFLS2, GI: 984880651) as queries, three separate TBLASTNs (protein query to translated (six frames) nucleotide database) against the Rutaceae family (taxid: 23513) were performed using the National Center for Biotechnology Information (NCBI) BLAST interface. Two contiguous genes were consistently identified as the most homologous genes in both *C. sinensis* and *C. clementina*. In *C. sinensis*, the two loci are LOC102618529 and LOC102608136 with amino acid sequence identity of 54 and 55% to AtFLS2, 60 and 61% to LeFLS2, and 62 and 63% to VvFLS2, respectively. The *C. clementina* genome also contains the two genes with corresponding locus names CICLE v10018646mg and CICLE v10024610mg. To simplify nomenclature from here on, LOC102618529/CICLE v10018646mg is referred to as *FLS2-1* and LOC102608136/CICLE v10024610mg as *FLS2-2* (Supplementary Table 1).

**Figure 1:**
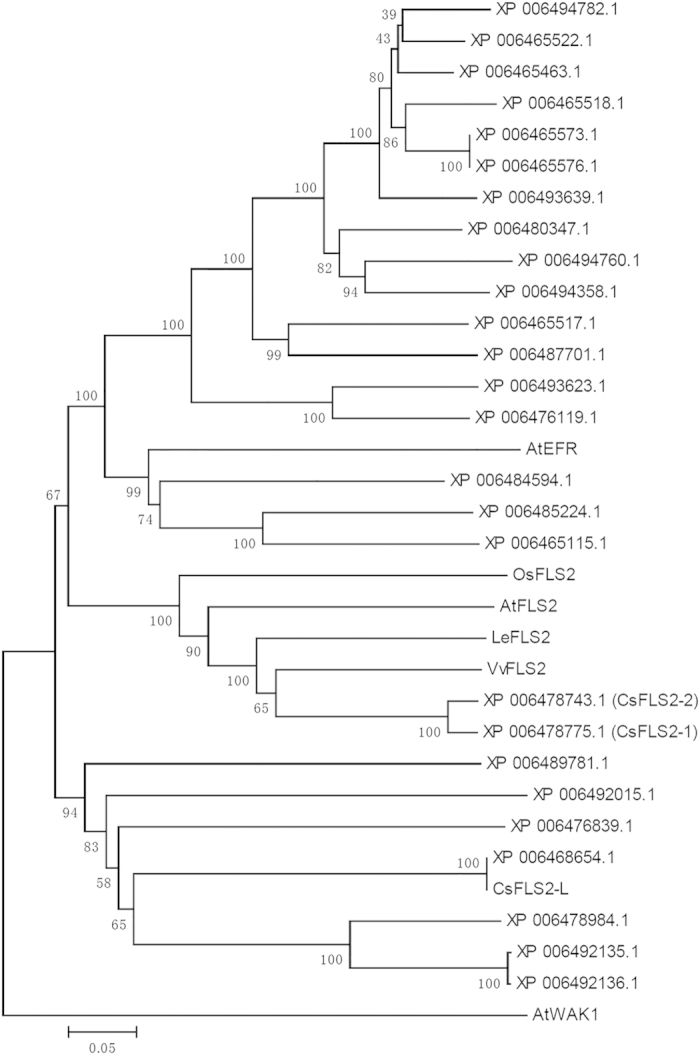
Neighbor-joining phylogenetic tree^47^ indicating evolutionary relationships between *C. sinensis* FLS2s (CsFLS2-1 and CsFLS2-2), previously reported CsFLS2-Like (CsFLS2-L),^22^ *Arabidopsis* (AtFLS2), tomato (LeFLS2), rice (OsFLS2) and grapevine (VvLFS2), different classes of receptor kinases (AtEFR and AtWAK1), and other homologous *C. sinensis* proteins obtained through BLAST search (using the AtFLS2 protein to BLAST against the *C. sinensis* database). The percentage of replicate trees in which the associated taxa clustered together in the bootstrap test (500 replicates) are shown next to the branches.^48^ The evolutionary distances were computed using the p-distance method and are in the units of the number of amino acid differences per site. The analysis involved 33 amino acid sequences. All positions containing gaps and missing data were eliminated. There were a total of 492 positions in the final data set. Evolutionary analyses were conducted in MEGA5.^49^

Phylogenetic analysis of 26 homologous protein sequences identified by a BLAST search in *C. sinensis* showed that CsFLS2-1 (XP_006478775.1) and CsFLS2-2 (XP_006478743.1) have the closest evolutionary distance to the FLS2 orthologs from *Arabidopsis* (AtFLS2), tomato (LeFLS2), rice (OsFLS2) and grapevine (VvFLS2), relative to other citrus proteins. These sequences are different from other characterized receptor-like kinases including EFR and WAK1 that mediate the perception of bacterial elongation factor Tu^28^ and cell wall-derived danger signal oligogalacturonide,^29,30^ respectively (Figure 1). It is worth noting that a recently reported citrus FLS2-like (CsFLS2-L) protein (orange1.1 g000859 from the Joint Genome Institute Phytozome database)^22^ was located in a distinct clade from all of the FLS2s (Figure 1). Located in tandem on the chromosome, the nucleotide sequence identity of the coding region for *CsFLS2-1* and *CsFLS2-2* is as high as 97%, suggesting they may originate from a gene duplication event. In addition, the structure of the two genes is highly similar to each other and to that of AtFLS2 (Supplementary Figure 1). Overall, the bioinformatics analysis supports that the two identified candidates are citrus *FLS2* flagellin receptor genes.

### Sequencing of the *FLS2* candidates from ‘Duncan’ grapefruit (*C. paradisi*), ‘Sun Chu Sha’ mandarin (*C. reticulata*) and ‘Nagami’ kumquat (*F. margarita*)

We first set out to compare the sequences of *FLS2* from ‘Nagami’ kumquat, ‘Sun Chu Sha’ mandarin and ‘Duncan’ grapefruit because they represent citrus species of different canker-resistant/-susceptible levels: highly resistant, moderately resistant and highly susceptible, respectively. Moreover, our previous work showed that they have different responsiveness to the Xflg22 treatment, suggesting FLS2-mediated PTI affects canker resistance in citrus.^23^

**Figure 2:**
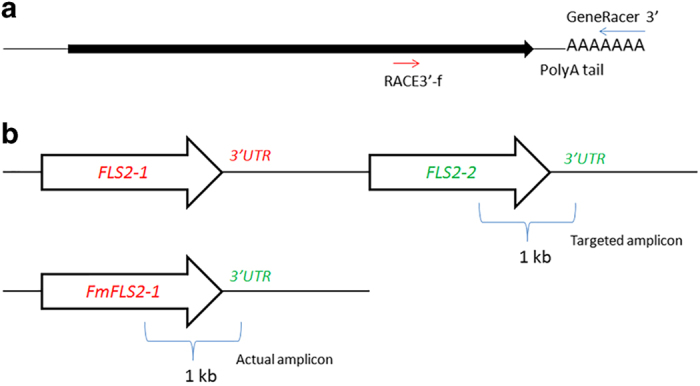
RACE PCR to attempt the amplification of *FmFLS2-2*. (**a**) Using ‘Nagami’ kumquat cDNA as template a forward primer (RACE3ʹ-f, Supplementary Table 1) was used in combination with the universal reverse primer (GeneRacer 3ʹ, Supplementary Table 1), which hybridizes to the poly-A tail at the 3ʹ end of the messenger RNA. (**b**) Schematic representation of the theoretical target versus the PCR product obtained. Top: based on the genomic map reported for *C. sinensis* and *C. clementina* (http://citrus.hzau.edu.cn/orange/ and Phytozome.org) the expected 1-kb-long amplicon would be composed of the 3ʹ protein-coding region of *FLS2-2* and its corresponding 3′ UTR. Bottom: the actual amplicon, as revealed by sequencing, was a combination of the protein-coding region of *FLS2-1* and the 3′ UTR of *FLS2-2.* cDNA, complementary DNA; UTR, untranslated region.

**Figure 3:**
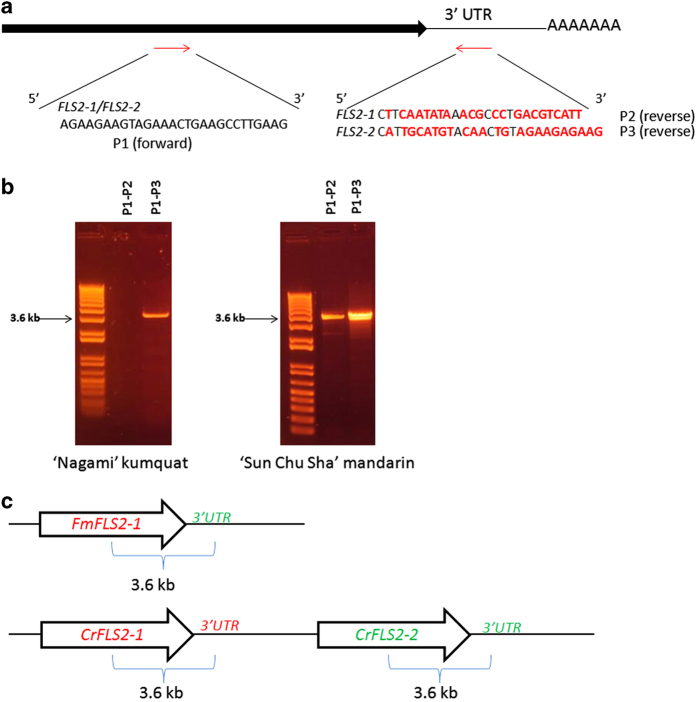
PCR amplification of the 3ʹ portions of *FLS2-1* and *FLS2-2* from ‘Nagami’ kumquat and ‘Sun Chu Sha’ mandarin. (**a**) Primers used in the PCR reactions. The forward P1 primer targets a sequence in the coding region that is identical in the two genes. The reverse primers P2 and P3 are specific to the 3′ UTRs of *FLS2-1* and *FLS2-2*, respectively. (**b**) PCR amplicons obtained on agarose gels. The combination of primers used is shown on top and the approximate size on the left. (**c**) Representation of the genomic organization and position of the amplicons obtained based on sequencing of the PCR products. Top: only the P1–P3 primer combination produced an amplicon in ‘Nagami’ kumquat. Sequencing also confirmed it contained a 3ʹ coding region similar to *FLS2-1* and a 3′ UTR similar to *FLS2-2*. Bottom: in ‘Sun Chu Sha’ mandarin both primer combinations (P1–P2 and P1–P3) produced the expected *FLS2-1* and *FLS2-2* sequences with their corresponding 3′ UTRs. UTR, untranslated region.

Using gene-specific primers, the two candidate genes were amplified and sequenced from ‘Sun Chu Sha’ mandarin (*CrFLS2-1* and *CrFLS2-2*) and ‘Duncan’ grapefruit (*CpFLS2-1* and *CpFLS2-2*). However, we could only amplify one gene from ‘Nagami’ kumquat (*FmFLS2-1*). Because kumquat is a more distant relative from sweet orange and clementine than mandarin and grapefruit, there was a possibility that primers based on these species would not work in kumquat due to potential greater sequence divergence. Hence an alternative PCR strategy was used to obtain the sequence of this gene. RACE is a PCR technique that requires only one gene-specific primer, in combination with a universal sequence that is a signature of mRNAs (for example, 3ʹ poly-A tail). Using RACE, we attempted to perform the amplification of the 3ʹ region of the *FmFLS2-2* transcript with an *FLS2-2* forward primer based on *C. sinensis* sequences (Figure 2a and Supplementary Table 2). Interestingly, we obtained a gene segment that was a combination of the *FmFLS2-1* protein-coding region and the 3ʹ untranslated region (3ʹ UTR) homologous to *FLS2-2* (Figure 2b). In a separate experiment, we performed conventional PCR using 3′ UTR-specific reverse primers in combination with a forward primer common to *FLS2-1* and *FLS2-2* to differentially amplify the two gene segments (Figure 3a) from ‘Nagami’ kumquat and ‘Sun Chu Sha’ mandarin. Only one primer combination resulted in amplification in ‘Nagami’ kumquat (Figure 3b). Sequencing of this amplicon confirmed it was a ‘hybrid’ *FmFLS2-1* protein-coding/FLS2-2 3′ UTR gene segment similar to the one obtained by RACE (Figure 3c). In ‘Sun Chu Sha’ mandarin, however, both *CrFLS2-1* and *CrFLS2-2* with their corresponding 3′ UTRs were amplified using the same primer combinations (Figures 3b and c). These results suggest the absence of the *FmFLS2-2* protein-coding region in the genome of ‘Nagami’ kumquat.

### Amino acid sequence examination of the putative citrus FLS2s confirms the existence of functional domains

**Figure 4:**
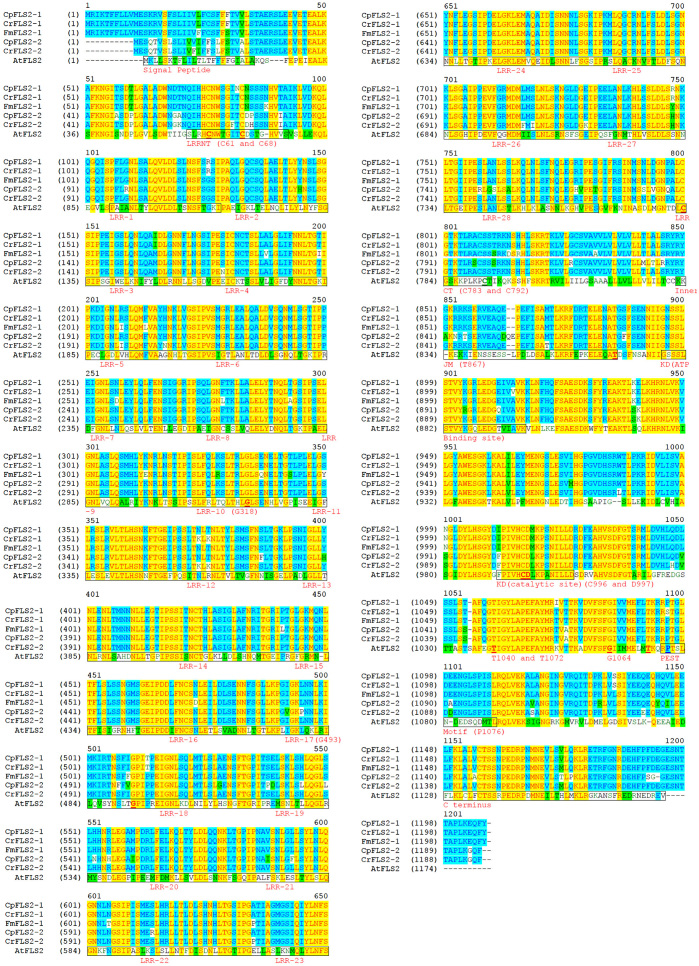
Protein sequence alignment of putative citrus FLS2s and AtFLS2. ‘Duncan’ grapefruit (CpFLS2-1 and CpFLS2-2), ‘Sun Chu Sha’ mandarin (CrFLS2-1 and CrFLS2-2), ‘Nagami’ kumquat (FmFLS2-1) and *Arabidopsis* *thaliana* (AtFLS2) were aligned using AlignX (Vector NTI package, Invitrogen). The functional domains were marked with black boxes and the residues with underlined bold letters. The critical amino acid positions were based on the AtFLS2 protein sequence. Inner JM, inner juxtamembrane; KD, kinase domain; LRR, leucine-rich repeat; LRRCT, LRR C-terminal domain; LRRNT, LRR N-terminal domain.^31,32^

Extensive studies on FLS2 function in other plants have been conducted and they provide useful information on important domains and residues that are conserved across plant species.^31,32^ To determine whether the putative citrus FLS2 proteins contain all the important motifs and amino acids, we compared predicted amino acid sequences of CrFLS2-1, CrFLS2-2, CpFLS2-1, CpFLS2-2 and FmFLS2-1 to that of AtFLS2 (Figure 4). Twenty eight LRRs along with their LRRNT and LRRCT^13,31^ were identified, based on homology with the LxxLxLxxN motif, in the sequences of all three citrus species (Figure 4). Critical amino acid residues including C61 and C68 in LRRNT, C783 in LRRCT, G318 in LRR-10 and G493 in LRR-17 were also found to be conserved,^13,33–35^ with the exception of a C to S substitution at position 792 (Figure 4). Within the kinase domain, the ATP-binding site, a catalytic site^36^ and the PEST motif^37^ were identified in the citrus FLS2s and residues including C996 and D997 (non-RD kinase),^38^ T1040 and T1072 (reactive oxygen species (ROS) production),^39^ G1064 (autophosphorylation)^13,40,41^ and P1076 (FLS2 ubiquitination and endocytosis)^37^ were also confirmed from the corresponding domains, except that there was an S in place of P at position 1076 in CrFLS2-1(Figure 4). The Inner JM^42^ of the citrus FLS2s was also identified with the confirmation of the residue T867;^39^ however, a two amino acid insertion (Q and E) within this domain was observed in CpFLS2-2 in comparison to the proteins from other genotypes (Figure 4). At the C terminus,^31^ the citrus FLS2 proteins contained a highly conserved region which aligned to the AtFLS2 C terminus, but there were five amino acid variations observed only in CpFLS2-2 (Figure 4).

### Citrus *FLS2-1* and *FLS2-2* are differentially regulated at the transcriptional level

**Figure 5:**
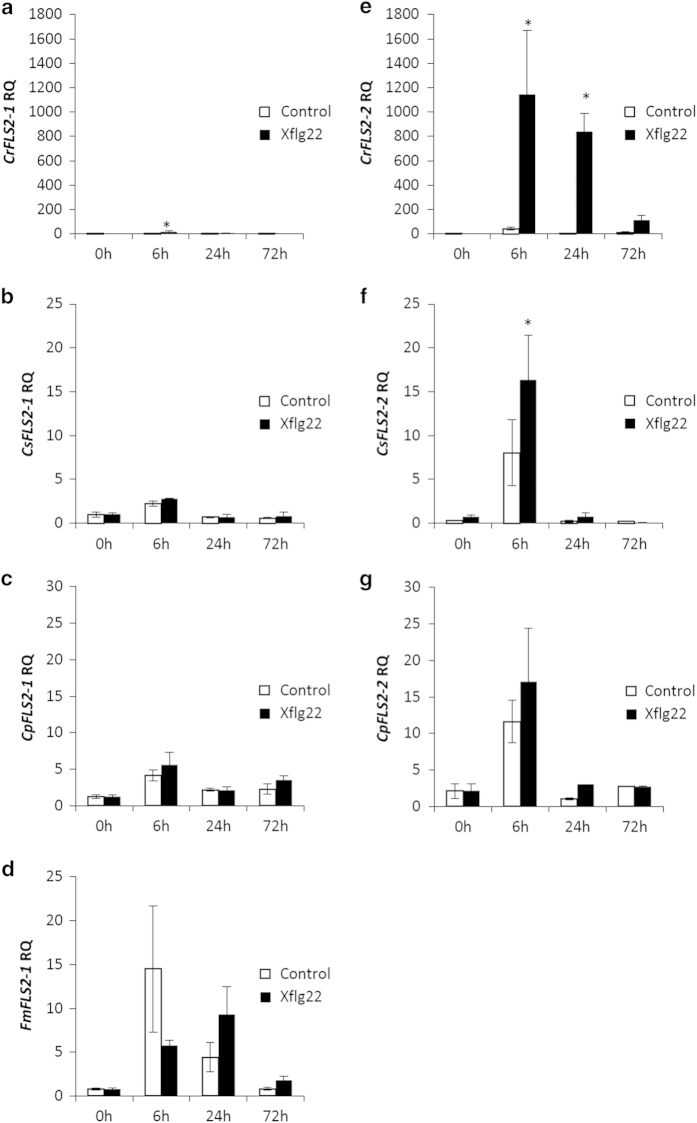
Effect of Xflg22 on the expression of the *FLS2* candidate genes in ‘Sun Chu Sha’ mandarin (**a** and **e**), ‘Navel’ sweet orange (**b** and **f**), ‘Duncan’ grapefruit (**c** and **g**) and ‘Nagami’ kumquat (**d**). RQ is the relative quantification of gene expression levels after water control (white) or 10 μm Xflg22 (black) infiltrations. One control treatment sample at the time 0 was selected as the reference for RQ calculations. An asterisk indicates RQ values significantly different (*P*<0.05) from pre-inoculation levels (0 h) and from the water controls at the particular time point. Bars are means of replicates±s.e. (*n*=3).

**Figure 6:**
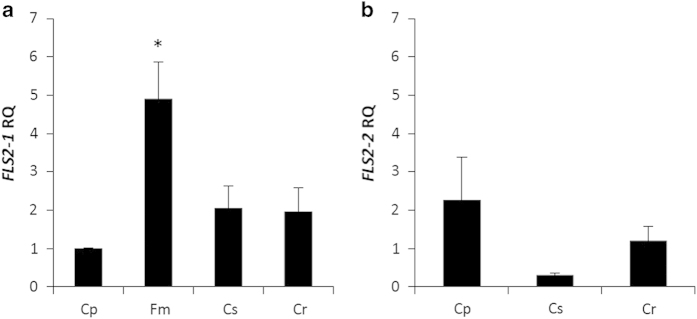
Steady-state expression levels of *FLS2-1* (**a**) and *FLS2-2* (**b**) in ‘Duncan’ grapefruit (Cp), ‘Nagami’ kumquat (Fm), ‘Navel’ sweet orange (Cs) and ‘Sun Chu Sha’ mandarin (Cr). RQ is the relative quantification of gene expression levels from young fully expanded leaves randomly collected from three replicated plants. One ‘Duncan’ grapefruit sample was selected as the reference for RQ calculations. Tukey multiple comparisons of means was used for statistical analysis. An asterisk indicates that the RQ value is significantly different (*P*<0.05) from other values. Bars are means of replicates±s.e. (*n*=3).

Specific primers and probes were designed for RT–qPCR to study the expression of the two *FLS2* candidates (Supplementary Table 3). To compare the induction pattern of the two genes, citrus plants were treated with either Xflg22 or water as the control. Subsequently leaf tissue was collected in a time course at 0, 6, 24 and 72 h after treatment. ‘Sun Chu Sha’ mandarin, ‘Navel’ sweet orange, ‘Duncan’ grapefruit and ‘Nagami’ kumquat were studied to compare the *FLS2* expression induced by Xflg22 between citrus canker-resistant and -susceptible species. The results showed that in the canker-resistant ‘Sun Chu Sha’ mandarin, *CrFLS2-1* was significantly induced at 6 h and *CrFLS2-2* was induced at 6 and 24 h by the Xflg22 treatment; however, the induction level of *CrFLS2-2* was several orders of magnitude higher than that of *CrFLS2-1* (Figures 5a and e). Similarly, in canker-susceptible ‘Navel’ sweet orange and ‘Duncan’ grapefruit, *CsFLS2-2* and *CpFLS2-2* were more highly induced by Xflg22 than *CsFLS2-1* and *CpFLS2-1*, although the induction levels were much lower than that of *CrFLS2-2* (Figures 5b, c, f and g). In ‘Nagami’ kumquat, Xflg22 did not significantly induce *FmFLS2-1* at the time points studied compared with the water controls, although there was an increase in *FmFLS2-1* expression in the control treatment at 6 h (Figure 5d). No expression of *FLS2-2* was detected in ‘Nagami’ kumquat with or without the Xflg22 treatment (data not shown), once again confirming that this species has only one *FLS2* gene. Also, the steady-state expression of *FLS2-1* and *FLS2-2* was measured from leaf tissue without any treatment. Interestingly, ‘Nagami’ kumquat had a significantly higher expression level of *FmFLS2-1* than the *FLS2-1* in ‘Duncan’ grapefruit, ‘Navel’ sweet orange and ‘Sun Chu Sha’ mandarin (Figure 6a), whereas there were no differences in *FLS2-2* expression between the citrus species tested (Figure 6b).

### Ectopic expression of FmFLS2-1 and CrFLS2-2 enhances Xflg22 responsiveness and canker resistance in susceptible ‘Duncan’ grapefruit

**Figure 7:**
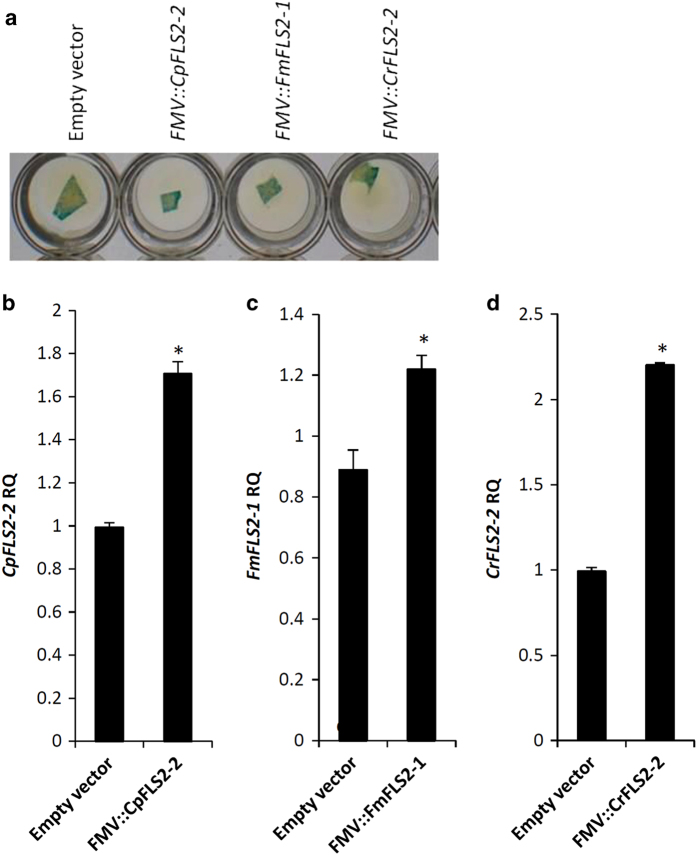
Evaluation of the transient expression using GUS assays (**a**) and RT–qPCR (**b**–**d**). Agroinfiltrated ‘Duncan’ grapefruit leaves were collected 3 days after infiltration and leaf segments were used in a GUS histochemical staining assay. Leaf segments were also evaluated for transgene expression using RT–qPCR. RQ values are the relative quantification of gene expression 3 days after agroinfiltration. An asterisk indicates RQ value of the transgene is significantly different (*P*<0.05) from the control (empty vector). Bars are means of replicates±s.e. (*n*=3). GUS, β-glucuronidase histochemical staining assay; RT–qPCR, quantitative reverse transcription–PCR.

**Figure 8:**
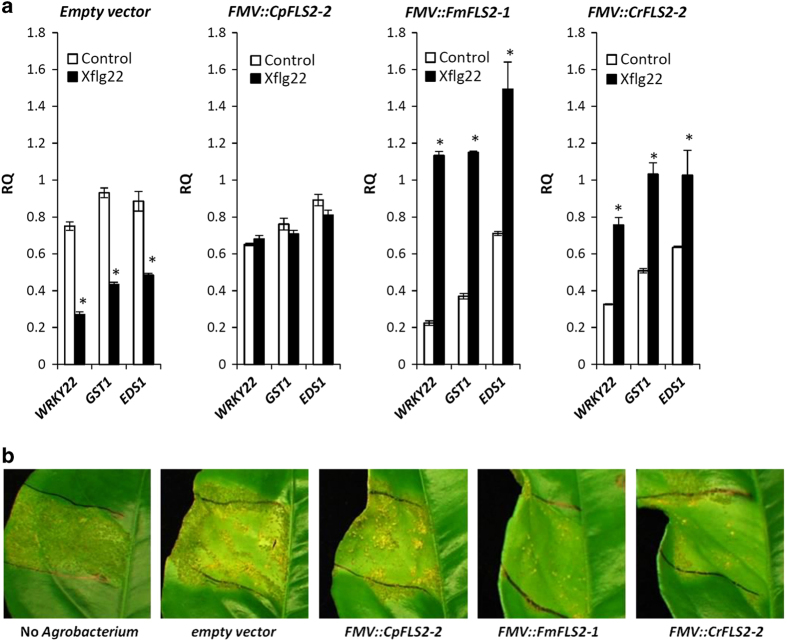
Response to Xflg22 and citrus canker in ‘Duncan’ grapefruit leaves transiently expressing various citrus FLS2 plasmid constructs. (**a**) Leaves were treated with 10 μm Xflg22 or water as the control 3 days after infiltration with *Agrobacterium* containing the plasmids. The leaves were tested for Xflg22-responsive gene expression 24 h after the treatment. The constructs tested are indicated above each graphic. RQ is the relative quantification of gene expression 24 h after Xflg22 or water (control) treatment. An asterisk represents RQ values significantly different (*P*<0.05) from the water control. Bars are means of replicates±s.e. (*n*=3). (**b**) *Xcc* bacterial suspensions (5×10^5^ c.f.u. per mL) were inoculated into leaves 1 day after the agroinfiltrations. The citrus canker symptoms were recorded 2 weeks after the inoculations. The plasmid constructs and controls used in the agroinfiltrations are indicated below. The experiments were repeated twice with similar results. c.f.u., colony-forming unit.

Our ultimate objective is to identify genes with good potential to increase Xflg22 responsiveness and bacterial resistance. Based on the results described above, we selected *CrFLS2-2* because it derives from the canker-resistant ‘Sun Chu Sha’ mandarin and it was the most highly induced gene by Xflg22. We also chose *FmFLS2-1* because ‘Nagami’ kumquat shows the highest level of resistance to citrus canker, despite the fact that it did not seem inducible by Xflg22 although it maintained the highest pre-induction expression level. In addition, we included *CpFLS2-2* to determine whether differences in protein sequence rather than expression levels affected the defense response. The *FLS2* genes were cloned into the *Agrobacterium* binary expression vector (pCAMBIA2201) under a constitutive promoter (FMV promoter) with a GUS gene as a reporter. Using leaf agroinfiltration, the genes were transiently expressed in ‘Duncan’ grapefruit, a citrus species that is weakly responsive to Xflg22 and highly susceptible to canker. Subsequently agroinfiltrated leaves were challenged with either Xflg22 or *Xcc* (Figures 7 and 8).

A histochemical GUS expression assay was used to evaluate the efficiency of the agroinfiltration. Blue staining was observed 3 days after the *Agrobacterium* infiltration for all of the constructs tested (Figure 7a). In addition, the expression of the transgenes was confirmed by RT–qPCR, as shown by significantly higher levels of *CpFLS2-2*, *FmFLS2-1* and *CrFLS2-2* than the control (empty vector) at 3 days after infiltration (Figures 7b–d).

The response to Xflg22 was determined by measuring the oxidative burst and induction of *WRKY22*, *GST1* and *EDS1*, three defense-related genes that were previously found to be highly responsive to Xflg22 treatment in citrus.^23^ There was no obvious elevation of ROS production in the leaves with *FMV::CpFLS2-2*, *FMV::FmFLS2-1* or *FMV::CrFLS2-2* relative to that of the empty vector control (Supplementary Figure 2). However, the measurement of Xflg22-responsive genes showed that *FMV::FmFLS2-1-* and *FMV::CrFLS2-2*-expressing leaves had significantly higher transcription levels of *WRKY22*, *GST1* and *EDS1* 24 h after the Xflg22 treatment compared with the water controls. Leaves inoculated with *Agrobacterium* containing an empty vector showed significantly lower expression of the three marker genes compared with the controls (Figure 8a). The *FMV::CpFLS2-2* construct did not cause differences in the induction of *WRKY22*, *GST1* and *EDS1* by the Xflg22 treatment (Figure 8a).

To determine if the transgene-mediated response had an effect on citrus canker resistance, we inoculated *Xcc* (5×10^5^ c.f.u.  per mL) into the leaves expressing the different transgenes. ‘Duncan’ grapefruit leaves containing *FMV::FmFLS2-1* and *FMV::CrFLS2-2* showed less symptoms, as indicated by the reduced number of canker lesions in those leaves when compared with leaves inoculated with the empty vector control (Figure 8b). On the other hand, expression of *FMV::CpFLS2-2* did not result in any obvious differences in symptom development when compared with the control (Figure 8b).

## Discussion

Using publically available genomic databases, we identified two *FLS2* candidate genes in the genomes of *C. sinensis* and *C. clementina* (Supplementary Table 1). Phylogenetic analysis showed that, among 26 citrus proteins found through BLAST search homologous to AtFLS2, only FLS2-1 and FLS2-2 were closely related to *bona fide* FLS2s from various plant species (Figure 1). The predicted gene structures of the two citrus FLS2s (number, position and size of exons/introns) were also similar to that of AtFLS2 (Supplementary Figure 1). Hence, the bioinformatics results strongly suggest we have identified the genes encoding the bacterial flagellin receptors in citrus. Based on the database sequences, we designed primers to amplify and sequence the two *FLS2* genes from ‘Duncan’ grapefruit (*CpFLS2-1* and *CpFLS2-2*) and ‘Sun Chu Sha’ mandarin (*CrFLS2-1* and *CrFLS2-2*). Our results indicate that the presence of two *FLS2* orthologs is probably conserved across the genus *Citrus*. *FLS2-1* and *FLS2-2* were also found in pummelo (*C. grandis*) and *Microcitrus australis*, a more distant relative of *Citrus* in the Rutaceae family (VJF, unpublished results). On the other hand, only one *FLS2* (*FmFLS2-1*) was obtained from ‘Nagami’ kumquat (*F. margarita*), and analysis of its flanking region indicated that the protein-coding sequence of *FmFLS2-2* may not exist in the genome of this species (Figures 2 and 3) although the 3′ UTR and terminator still remain. To confirm the copy number of *FLS2* in kumquat, additional approaches such as Southern blot analysis will have to be performed; however, this was beyond the scope of our study.

Comparison of the predicted citrus FLS2 amino acid sequences with the well-characterized AtFLS2 show that CpFLS2-1, CpFLS2-2, CrFLS2-1, CrFLS2-2 and FmFLS2-1 contain the essential domains that are the hallmark of this protein.^31^ These domains are: the extracellular 28 LRRs, LRRNT, LRRCT, the intracellular ATP-binding site, the catalytic site, the PEST motif, the inner JM and a conserved C terminus (Figure 4). The presence of these domains in the citrus protein sequences is another strong indication that the candidate genes are flg22 receptors. In addition, confirmation of critical amino acids in the citrus proteins (Figure 4) implies they potentially have receptor-like kinase activity including ligand recognition and binding,^13,35,43^ non-RD kinase catalysis,^38^ kinase autophosphorylation^13,40,41^ and flg22-triggered oxidative burst.^39^ However, there were amino acid variations between some of the citrus FLS2s and AtFLS2, and also between citrus proteins. Examples are the substitution of P to S at position 1076 in the PEST motif of CrFLS2-1, a two amino acid insertion (QE) within the inner JM domain and a five amino acid variation in the conserved C terminus of CpFLS2-2 (Figure 4). Whether these amino acid alternations have an effect on function remains to be determined.

Xflg22 treatment induced higher levels of *FLS2-2* expression relative to the control than *FLS2-1* in ‘Sun Chu Sha’ mandarin, ‘Navel’ sweet orange and ‘Duncan’ grapefruit (Figure 5), indicating FLS2-2 is the more responsive receptor to the flg22 elicitor in these citrus genotypes. It has been reported in *Arabidopsis* that the higher accumulation of *AtFLS2* transcripts correlates with a stronger flg22-induced oxidative burst.^44^ Moreover, the fold change induced by Xflg22 on *FLS2-2* was much higher and induction time was longer in ‘Sun Chu Sha’ mandarin than in ‘Navel’ sweet orange and ‘Duncan’ grapefruit, indicating that the high and long lasting expression of this gene correlated with the reported levels of ROS production and citrus canker resistance.^23^ On the other hand, *FLS2-1* from ‘Nagami’ kumquat showed a low level of induction by Xflg22 but the highest pre-induction expression level among the citrus species tested (Figures 5d and 6a). In addition, no expression of *FmFLS2-2* was ever detected, indicating the absence of transcript, which is consistent with our interpretation that this gene is not fully present in the genome of ‘Nagami’ kumquat (the RT–qPCR primers and probes used target the coding regions). In *Arabidopsis*, evidence has shown that FLS2 belongs to a ‘preexisting recognition system’ where its steady-state expression associates with flg22-triggered defenses.^13^ The high *FLS2-1* transcriptional level observed in ‘Nagami’ kumquat in untreated leaves suggests this citrus species maintains high FmFLS2-1 protein abundance which allows it to achieve a quick and strong Xflg22 response. It will be interesting to determine whether the native promoter of *FmFLS2-1* drives a stronger basal expression compared with those from other citrus species, and whether it is highly inducible by other signaling cues such as hormones, wounding and so on.^45^ Such a study may help understand how ‘Nagami’ kumquat evolved a robust PTI that is deemed essential to canker resistance.^23^ In addition, other defense mechanisms including the accumulation of phytoalexins and/or terpenoids cannot be ruled out as having an important role in the observed high level of resistance to citrus canker in ‘Nagami’ kumquat.

When disease resistance genes are to be integrated into commercial crop cultivars using genetic transformation methods, the use of genes from the same species or cross-compatible species (cisgenesis) is often preferred over those from unrelated species (transgenesis).^46^ Using an *Agrobacterium*-mediated gene transient expression system, we tested *FmFLS2-1*, *CrFLS2-2* and *CpFLS2-2* for their efficacy to enhance *Xcc* resistance. The Xflg22 treatment resulted in higher inductions of *WRKY22*, *GST1* and *EDS1* in leaves with ectopic expression of *FMV::FmFLS2-1* and *FMV::CrFLS2-2* (Figure 8a). Consistently, when inoculated with *Xcc*, ‘Duncan’ grapefruit leaves expressing *FMV::FmFLS2-1* and *FMV::CrFLS2-2* showed less symptoms relative to leaves inoculated with an empty vector and those with *FMV::CpFLS2-2* (Figure 8b). These results indicate the transient expression method was effective in citrus for quickly testing transgenes for their ability to enhance host defense and provide pathogen protection without the use of a lengthy and labor-intensive stable transformation procedure. We also show that *FmFLS2-1* and *CrFLS2-2* are such capable genes and conclude that *FmFLS2-1* and *CrFLS2-2* encode functional bacterial flagellin/flg22 receptors involved in the perception of the PAMP and induction of the PTI response. Conversely, the ectopic expression of CpFLS2-2 did not result in the induction of PTI/defense marker genes nor increased resistance to *Xcc*. 'Duncan’ grapefruit is susceptible to citrus canker and it is thus possible that the CpFLS2-2 protein contains mutations (Figure 4) that compromise the activation of downstream defenses against *Xcc*. Another possibility is that the transient expression method did not produce enough protein to initiate and enhance immunity, although this seems less likely based on the transcript levels detected experimentally (Figures 7b–d). Hence other genetic approaches need to be used to clarify whether CpFLS2-2 encodes or not a potent flg22 receptor and if the PTI triggered by this gene has a dose-dependent effect. Nevertheless, our survey of citrus *FLS2*s from different species along with transient expression tests identified *FmFLS2-1* and *CrFLS2-2* as potential resources to enhance PTI and achieve canker resistance in susceptible citrus species. In conclusion, the genomes of most citrus species contain two contiguous gene copies of the flagellin PRR receptor FLS2. Both copies seem functional and able to initiate PTI when derived from resistant genotypes but only one, FLS2-2, is induced by Xflg22. Pre-existing protein levels and amino acid sequence probably play important roles in determining the outcome of the citrus-*Xcc* interaction, that is, resistance versus susceptibility.
