# Supplementary material for: A survey of FLS2 genes from multiple citrus species identifies candidates for enhancing disease resistance to Xanthomonas citri ssp. citri
Source: Hortic Res. 2016 May 11;3:16022–. doi: 10.1038/hortres.2016.22 (PMC4863249; doi:10.1038/hortres.2016.22)
Supplement: Supplementary Information [file hortres201622-s1.pdf]

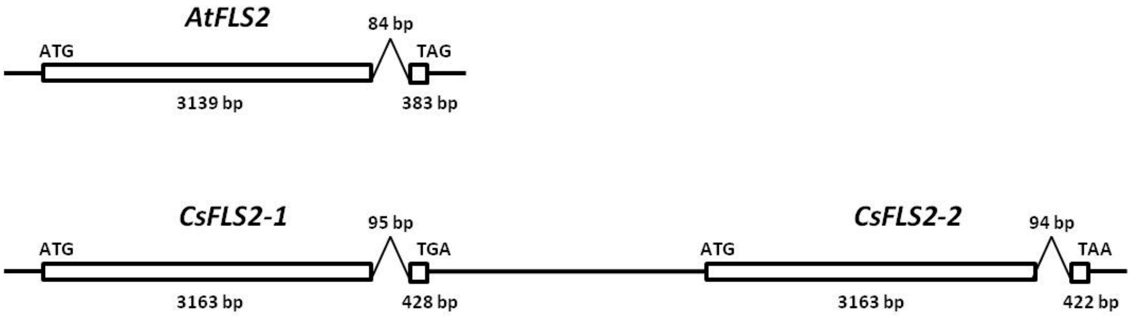

Supplementary Figure 1. Predicted gene structure of *CsFLS2-1* and *CsFLS2-2* compared to *AtFLS2*. The Boxes and folding lines represent exons and introns, respectively, with their length in base pair (bp). The straight lines stand for gene flanking regions. The start and stop codons are marked at the ends of each gene.

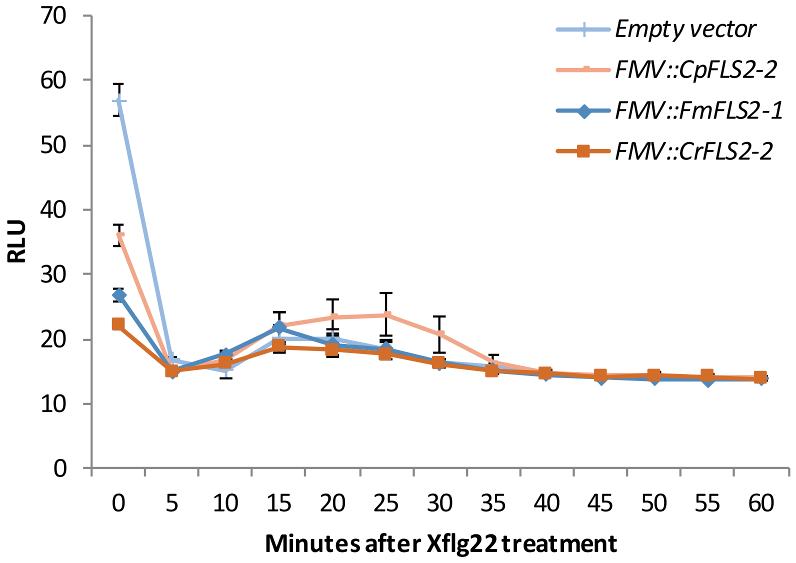

Supplementary Figure 2. Evaluation of Xflg22-triggered oxidative burst in 'Duncan' grapefruit transiently expressing *FMV::CpFLS2-2*, *FMV::FmFLS2-1* or *FMV::CrFLS2-2*. Agroinfiltrated leaves were collected 3 days after infiltration. Reaction solutions containing 100 nM Xflg22 were added to citrus leaf discs at minute 0. Relative light unit (RLU) was measured every 5 minutes for 60 minutes after the treatment. Values are means ± standard error (n= 15).

Supplementary Table 1. BLAST search using AtFLS2 protein as the query against citrus genomic databases identified two candidate genes named as *FLS2-1* and *FLS2-2*. The two genes were confirmed in the genome of both *C. sinensis* and *C. clementina.* Information on gene locus name, protein ID, annotation and genome submitter is shown in the table.

**Citrus species**	**Name given**	**Gene locus**	**Protein ID**	**Annotation**	**Submitter**
C. sinensis	*CsFLS2-1*	LOC102618529	XP_006478775.1	*C. sinensis* LRR receptor-like serine/threonine-protein kinase FLS2-like	China sweet orange genome project
	CsFLS2-2	LOC102608136	XP_006478743.1	*C. sinensis* LRR receptor-like serine/threonine-protein kinase FLS2-like	
C. clementina	*CcFLS2-1*	CICLE_v10018646mg	XP_006442977.1	*C. clementina* hypothetical protein	International Citrus Genome Consortium
	CcFLS2-2	CICLE_v10024610mg	XP_006442975.1	*C. clementina* hypothetical protein	

Supplementary Table 2. Primers for the PCR amplification of the citrus *FLS2* candidate genes.

Name	Target	Sequence	Remarks
VF395	*FLS2-2*	ATGGAGTCTCAAACAGTCAGCTTAAG	Forward primer, with start codon underlined.
VF396	*FLS2-2*	TTAGAACTGTCCTTTTAAAGGCGCTGTA	Reverse primer, with stop codon underlined.
VF397	*FLS2-1*	ATGAGGATCAAGACTTTTTTCCTTCTAGT	Forward primer, with start codon underlined.
VF399	*FLS2-1*	TCAATAGAACTGTTCTTTTAAAGGCGCTG	Reverse primer, with stop codon underlined.
QS1	*FLS2-1*<br>*FLS2-2*	TCAATTCCAGTTTCCATGGGTAGATTAG	Internal forward primer for sequencing of both *FLS2* candidate genes
QS2	*FLS2-1*<br>*FLS2-2*	TCTGTCTAAGAGAATATTGGAAGGCTTC	Internal reverse primer for sequencing of both *FLS2* candidate genes
QS3	*FLS2-1*<br>*FLS2-2*	GAGTGAAAACAATTTCAGTGGACTGCTG	Internal forward primer for sequencing of both *FLS2* candidate genes
RACE3'-f	*FLS2-2*	TTATCGAAGCTGAAGCACAGGAATCTG	Forward primer for 3' RACE
GeneRacer 3'	mRNA	GCTGTCAACGATACGCTACGTAACG	Universal reverse primer for 3' RACE
P1	*FLS2-1*<br>*FLS2-2*	AGAAGAAGTAGAAACTGAAGCCTTGAAG	Internal forward primer for both *FLS2* candidate genes
P2	*FLS2-1*	AATGACGTCAGGGCGTTTATATTGAAG	Reverse primer unique to the 3' UTR of *FLS2-1*
P3	*FLS2-2*	CTTCTCTTCTACAGTTGTACATGCAATG	Reverse primer unique to the 3' UTR of *FLS2-2*

Supplementary Table 3. List of RT-qPCR primers and probes. Full-length cDNA sequences of the *FLS2-1* and *FLS2-2* from 'Duncan' grapefruit and 'Sun Chu Sha' mandarin, *FLS2-1* from 'Nagami' kumquat and genomic sequences from 'Navel' sweet orange were used to design specific primers and probes. Sequences for *WRKY22*, *GST1* and *EDS1* have been described in a previous study. Forward primer (f), reverse primer (r) and probe (p) sequences were generated using Primer Express Software 3.0 (Applied Biosystems, Foster).

Target	Primer/Probe sequence
FLS2-1	f: AATGGCATCACCAGTGACACTT<br>r: CCAGACCAGTTGCAGTGATGA<br>p: AGGAGCACTTGCCG
FLS2-2	f: CCAAGCTCTCTGCGGAACA<br>r: AATGGTGTGAGTTTTTCCTTGATG<br>p: ACTCTCAGATCATGCAGC
WRKY22	f: GCGGATTGTCTCGCATGTG<br>r: TTATGGGTTTCTGCCCGTATTT<br>p: AAGTGGGCTTGGCG
GST1	f: GCCCGTTTGTCTCAGTCCAA<br>r: TGCAAATCGACCAAGGTGAA<br>p: ACTTGGCGTGCGACAG
EDS1	f: GGCTCGAGTATGCCCTGAAG<br>r: CTTGCCCAGAAACATGATTCC<br>p: ATCGGCAGGATCCAG
